# A rare presentation of childhood interstitial lung disease attributed to KDM3B gene mutation: a case report

**DOI:** 10.11604/pamj.2023.46.84.41457

**Published:** 2023-11-13

**Authors:** Zaineb Benslimane, Sinan Yavuz, Nader Francis

**Affiliations:** 1 General Pediatrics Department, Al Qassimi Women and Children Hospital, Sharjah, United Arab Emirates,; 2 Pediatric Pulmonology Department, Al Qassimi Women and Children Hospital, Sharjah, United Arab Emirates

**Keywords:** Children interstitial lung disease (chILD), pulmonary artery hypertension, KDM3B mutation, SIN3A mutation, case report

## Abstract

Childhood Interstitial Lung Disease (chILD) encompasses various respiratory conditions affecting children's lung airspaces and tissues, with diverse causes. One rare cause involves structural vascular changes. We describe a case of a 10-year-old boy diagnosed with chILD who exhibited specific dysmorphic features, developmental delay, and intellectual disability. He was diagnosed with severe pulmonary arterial hypertension (PAH) due to venous thromboembolic disease, an unusual underlying condition for chILD. A Whole Exome Sequence showed mutations in KDM3B and SIN3A genes, respectively responsible for Diets-Jongmans syndrome (DIJOS) and Witteveen-Kolk syndrome (WITKOS). Both syndromes can explain our patient´s phenotype and KDM3B mutation has been previously described to be associated with PAH. Our case suggests a potential association between KDM3B mutation and PAH leading to chILD. It also enriches the knowledge of genotypic diversity in KDM3B and SIN3A genes as well as the spectrum of clinical associations with DIJOS and WITKOS syndromes.

## Introduction

Childhood Interstitial Lung Disease (chILD) refers to a rare and diverse group of respiratory diseases of the lungs´ airspaces and tissues in children that differs from Interstitial Lung Disease seen in adults [1]. The diagnosis of chILD can be challenging, and many cases can be misdiagnosed. In fact, the spectrum of etiologies is vast and heterogeneous, covering among others infectious, innate, genetic and inflammatory origins [2]. Moreover, it has been shown that chILD can be associated with structural vascular changes like pulmonary hypertension or pulmonary veno-occlusive disease [3]. We report the case of a child with an atypical presentation of chILD with underlying severe pulmonary arterial hypertension (PAH) secondary to venous thromboembolic disease (chronic thromboembolic pulmonary hypertension). associated with mutations in both KDM3B and SIN3A genes.

## Patient and observation

**Patient information:** a 10-year-old male of Sudanese origin, who was initially diagnosed as a case of refractory asthma. He was frequently admitted to the hospital in view of recurrent episodes of respiratory distress with wheezing not responding to classical asthma treatment including systemic inhaled corticosteroids. His past medical history was significant for failure to thrive, congenital heart disease (Atrial Septal Defect, Ventricular Septal Defect and a Patent Ductus Arteriosus operated at 4 months of age) and transitory hypothyroidism for few months. His family history was positive for parental consanguinity as his parents were double second cousins. His father had an unspecified respiratory disorder. One sibling had pulmonary hypertension. His mother and his maternal aunt had miscarriages. Three of his maternal aunts had deep vein thrombosis and pulmonary embolism. One maternal aunt had valvopathy.

**Figure 1:**
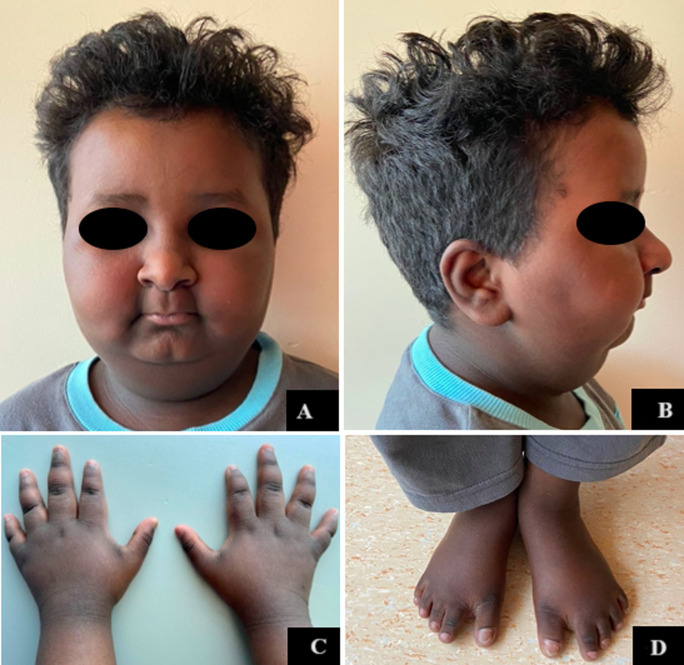
clinical photographs of our patient showing specific dysmorphic features; A) long face, broad and high forehead, hypertelorism, almond-shaped eyes, mildly down slanted palpebral fissures, broad nasal tip, long philtrum, thin upper lip, small mouth, B) micrognathia and short neck; extremities, C) brachydactyly, tapered fingers, clinodactyly of the 5^th^ finger, D) gap between first and second toe

**Clinical findings:** on examination, he was found to have short stature, obesity (BMI= 22.5), intellectual disability and specific physical features. His facial features included a long face, broad and high forehead, hypotelorism, almond-shaped eyes, mildly down slanted palpebral fissures, broad nasal tip, long philtrum, thin upper lip, small mouth and micrognathia. He also had a short neck, widely spaced and inverted nipples and dysmorphic extremities with brachydactyly, tapered fingers, straight ulnar side of the palm, clinodactyly of the 5^th^ finger and a gap between first and second toe (Figure 1).

**Timeline of current episode:** September 2021: echocardiography, CT scan and screening for auto-immune disease were performed. December 2021: diagnostic cardiac catheterization for pulmonary hypertension. January 2022: The whole exome sequence was sent. February 2022: planned for bronchoscopy with broncho-alveolar lavage and lung biopsy. October 2022: Pulmonary Perfusion Scan and thrombophilia work-up were done. He was admitted to the hospital multiple times from September 2021 with respiratory distress and increase of oxygen requirements.

**Figure 2:**
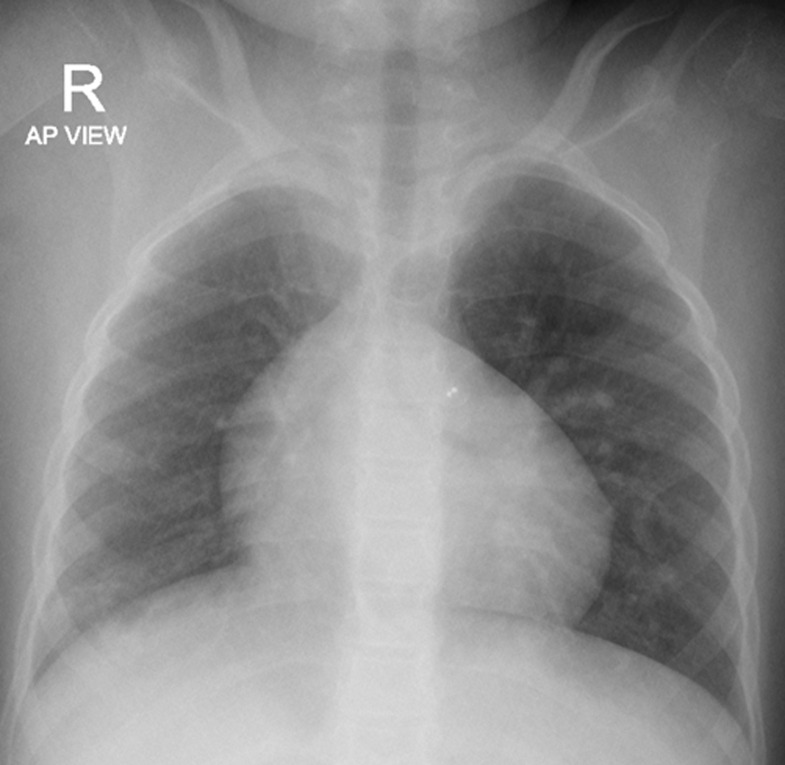
chest X-ray (antero-posterior view)

**Figure 3:**
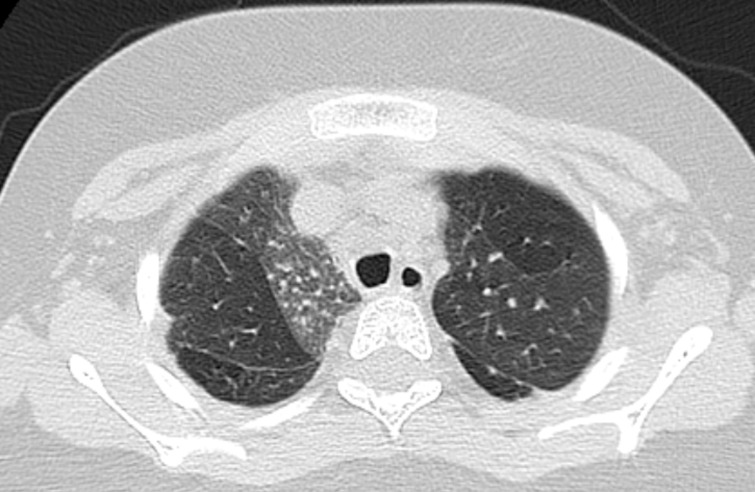
high resolution computed tomography of the chest (axial view)

**Figure 4:**
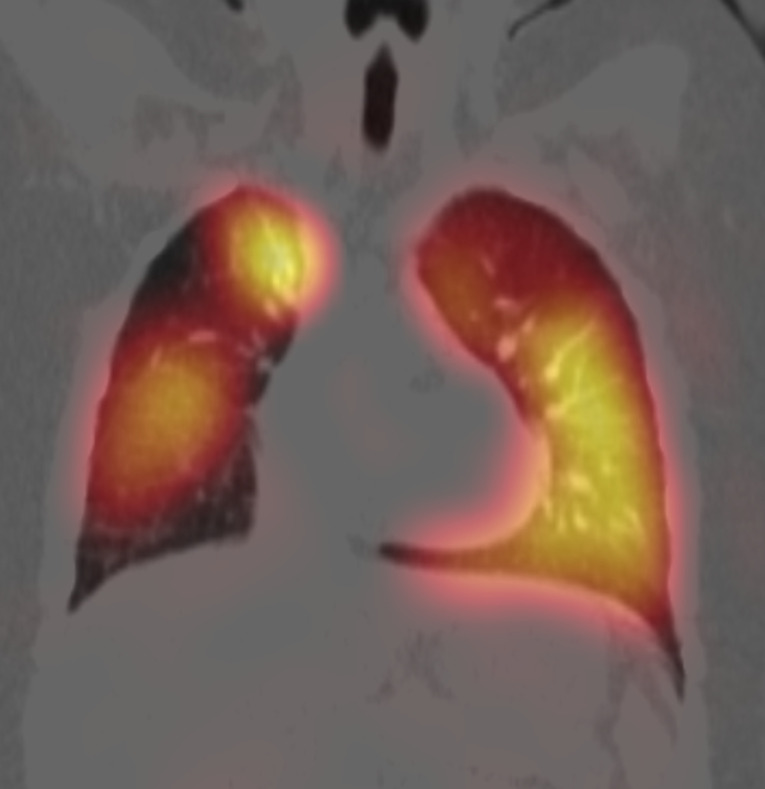
perfusion scan (coronal view)

**Diagnostic assessment:** serial chest X-rays were done throughout his hospital admissions and were consistently showing cardiomegaly, hyperinflation of the lungs, interstitial image and poor peripheral perfusion (Figure 2). His complex clinical and radiological presentation guided us to perform further investigations with the suspicion of chILD. A high-resolution CT chest showed a picture suggestive of interstitial lung disease with ground glass opacities, mosaic attenuation, fibrosis and hypoperfusion of both lungs’ fields (Figure 3). An echocardiography found severe PAH with mild tricuspid regurge, right ventricular hypertrophy, a Patent Foramen Ovale with mainly left to right shunt, mild to moderate right ventricular systolic and diastolic dysfunction as well as severe left ventricular diastolic dysfunction. A diagnostic cardiac catheterization for pulmonary hypertension was performed and confirmed the diagnosis. Finally, a Pulmonary Perfusion Scan (without ventilation) was done, and the images were suggestive for chronic thromboembolic disease with multiple perfusion defects in both lungs (Figure 4). In parallel with radiological investigations, a complete biological and genetic assessment were carried out looking for an underlying etiology. Initial laboratory tests were normal, including CBC, CRP, ESR, LFT, electrolytes and a coagulation profile. A screening for auto-immune disorders was also normal including total immunglubulins (IgA, IgM, IgG), antibodies (ANA, c-ANA, anti-ds DNA, p-ANCA, RF, tTg-IgA), Hepatitis B and C screening, TSH, PTH, C-peptide, C3 and C4. Finally, all thrombophilia work-up was done and reported normal. We intended to perform a bronchoscopy with broncho-alveolar lavage and lung biopsy but the patient developed severe pulmonary hypertension crises during the procedure that was cancelled. Finallly, a Whole-Exome Sequencing (WES) analysis to look for genetic causes of chILD was ordered. It identified two autosomal dominant heterozygous variants in the KDM3B and SIN3A genes. The KDM3B gene variant identified was c.4406T > Cp.(lle1469Thr) chr5: 137761266 and the SIN3A gene variant identified was c.1381G>A p.(Gly461Arg) chr15: 75699422. Both variants were not found in the public databases and were predicted to be pathogenic through in silico programs.

**Diagnosis:** the results were consistent with chILD associated with vascular changes due to severe PAH secondary to chronic thromboembolic disease along with a genetic confirmation of mutations in KDM3B and SIN3A genes.

**Therapeutic interventions:** he received monthly pulse therapy with Methylprednisolone 20 mg/kg for six months but there was no improvement so it was switched to daily oral Prednisolone 0,5 mg/kg/day. His treatment also included hydroxychloroquine 10 mg/kg, bosentan, sildenafil and inhalers of budesonide-formoterol and ipratropium.

**Follow-up and outcome of interventions:** we planned to repeat the bronchoscopy with broncho-alveolar lavage and lung biopsy, but the parents refused due to the child's prior episode of crisis. Despite treatment, our patient´s clinical condition deteriorated and he was admitted to the hospital more frequently with respiratory distress and increase in oxygen requirement.

**Informed consent:** the patient´s mother who is his legal guardian gave informed consent.

## Discussion

Diagnosis of chILD can be challenging, and it is not uncommon that cases get misdiagnosed like our patient who was initially believed to have asthma. This is due to the large variety of chILD etiologies and the non-specific nature of its presentation as well its low prevalence of likely < 1 per 100000 [2]. In fact, most centers will see maximum of 5 cases per year [2].

The diagnosis of chILD relies on noninvasive methods such as clinical history, chest X-ray, high-resolution CT scan, and genetic testing as well as invasive methods including bronchoalveolar lavage and transbronchial biopsy [3,4]. Genetic testing has significantly contributed to elucidate the large spectrum of chILD etiologies. In fact, up to 20% of chILD cases were found to be associated with genetic alterations like ABCA3, COPA, FLNA and FOXF1 genes mutations [3]. Consequently, the indication for genetic testing in cases of chILD should be made as early as possible, especially when patients present with strong family history, dysmorphic features or intellectual disability like our patient.

Our case is unique on two levels. Firstly, by its rare etiology found to be PAH that falls under the category of “ILD with structural vascular changes” of chILD´s classification proposed by the chILD-EU Network [3]. Secondly, by the discovery of two genetic mutations known to be responsible for Diets-Jongmans syndrome and Witteveen-Kolk syndrome.

Pathogenic variants in the KDM3B gene are causative for autosomal dominant Diets-Jongmans syndrome (DIJOS) [5]. KDM3B is part of an important group comprising the histone lysine methylases (KMTs) and histone lysine demethylases (KDMs) [5]. DIJOS syndrome is characterized by intellectual disability, developmental delay, short stature and facial dysmorphic features including broad nasal tip, low-hanging columella and thin upper lip [5]. These features were also found in our patient. Moreover, one case of a child with PAH was reported to be associated with a missense variant in the KDM3B gene [6], making our patient the second described case worldwide to the best of our knowledge.

Pathogenic variants in the SIN3A gene are causative for autosomal dominant Witteveen-Kolk syndrome (WITKOS) [7]. WITKOS syndrome is characterized by intellectual disability, developmental delay and a specific phenotype. Facial features include a long face, broad and high forehead, depressed nose bridge, mildly down slanted palpebral fissures and a small mouth [7] that were found in our patient. In their study, Witteveen *et al*. found an association with SIN3A gene and corticogenesis [7]. SIN3A was described to be an important factor of mammalian cerebral cortex development that could explain the intellectual disability and developmental delay [7].

Treatment for chILD varies based on the underlying cause. It usually includes corticosteroids, immunosuppressants like hydroxychloroquine, and supportive therapies [3]. Our patient was treated with corticosteroids, hydroxychloroquine, treatment of PAH with bosentan and sildenafil, oxygen support and symptomatic treatment with ipratropium and budesonide nebulizers. Despite treatment, his clinical condition deteriorated. It is well known in the literature that chILD represents a significant cause of morbidity and mortality, with an overall mortality rate of 15% [8].

## Conclusion

We report the case of a 10-year-old boy with chILD associated with vascular changes due to severe PAH secondary to chronic thromboembolic disease along with a genetic confirmation of mutations in KDM3B and SIN3A genes responsible for DIJOS syndrome and WITKOS syndrome. He exhibited many clinical features described in these syndromes and is, to the best of our knowledge, the second case of PAH associated with KDM3B gene mutation described in the literature. Our case suggests a possible association between KDM3B mutation and PAH leading to chILD. This case confirms the importance of thorough history, with a special focus on the family history, clinical examination and investigations to establish the diagnosis of chILD. It also highlights the value of considering multiple genetic causes in patients with complex phenotypes and enriches the knowledge of the genotypic diversity of the KDM3B and SIN3A variants as well as the spectrum of clinical associations with DIJOS and WITKOS syndromes.
